# Modeling the Human Visuo-Motor System to Support Remote-Control Operation

**DOI:** 10.3390/s18092979

**Published:** 2018-09-06

**Authors:** Jonathan Andersh, Bérénice Mettler

**Affiliations:** 1 Department of Computer Science and Engineering, University of Minnesota, Minneapolis, MN 55455, USA; ande1020@umn.edu; 2 International Computer Science Institute (ICSI), Berkeley, CA 94704, USA

**Keywords:** visuo-motor, teleoperation, human–machine interface

## Abstract

The working hypothesis in this project is that gaze interactions play a central role in structuring the joint control and guidance strategy of the human operator performing spatial tasks. Perceptual guidance and control is the idea that the visual and motor systems form a unified perceptuo-motor system where necessary information is naturally extracted by the visual system. As a consequence, the response of this system is constrained by the visual and motor mechanisms and these effects should manifest in the behavioral data. Modeling the perceptual processes of the human operator provides the foundation necessary for a systems-based approach to the design of control and display systems used by remotely operated vehicles. This paper investigates this hypothesis using flight tasks conducted with remotely controlled miniature rotorcraft, taking place in indoor settings that provide rich environments to investigate the key processes supporting spatial interactions. This work also applies to spatial control tasks in a range of application domains that include tele-operation, gaming, and virtual reality. The human-in-the-loop system combines the dynamics of the vehicle, environment, and human perception–action with the response of the overall system emerging from the interplay of perception and action. The main questions to be answered in this work are as follows: (i) what is the general control and guidance strategy of the human operator, and (ii) how is information about the vehicle and environment extracted visually by the operator. The general approach uses gaze as the primary sensory mechanism by decoding the gaze patterns of the pilot to provide information for estimation, control, and guidance. This work differs from existing research by taking what have largely been conceptual ideas on action–perception and structuring them to be implemented for a real-world problem. The paper proposes a system model that captures the human pilot’s perception–action loop; the loop that delineates the main components of the pilot’s perceptuo-motor system, including estimation of the vehicle state and task elements based on operator gaze patterns, trajectory planning, and tracking control. The identified human visuo-motor model is then exploited to demonstrate how the perceptual and control functions system can be augmented to reduce the operator workload.

## 1. Introduction

**Figure 1:**
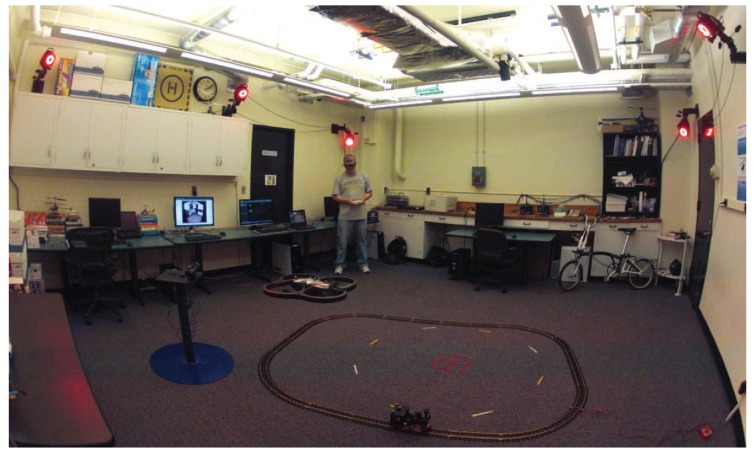
Lab facility for investigating human perception–action using a miniature unmanned aerial vehicle (UAV) with motion and gaze sensing.

Recent years have seen rapid advances in fields such as robotics and sensor technology that are fundamentally changing the way in which humans interact with the world. Improved robotics technology has led to an expanding number of applications that range from self-driving cars [1,2], to robotic-assisted surgery [3], and further to the wide availability of small-scale unmanned aerial vehicles [4]. At the same time, sensor capabilities have advanced and can provide inexpensive measurements of human gaze and body motion. Combining these technologies allows for the investigation of human performance while conducting tasks requiring human–machine interaction. Figure 1 shows a robotic unmanned aerial vehicle (UAV) operating in the Interactive Guidance and Control (IGCL) lab where operator gaze and motion can be measured. A systematic modeling approach utilizes the data captured from experimental flight tests to characterize the human pilot’s interaction with the vehicle and environment. The primary objective of the work that follows is to utilize the pilot’s gaze to model the human perception–action processes and implement augmentations for a teleoperation configuration.

**Figure 2:**
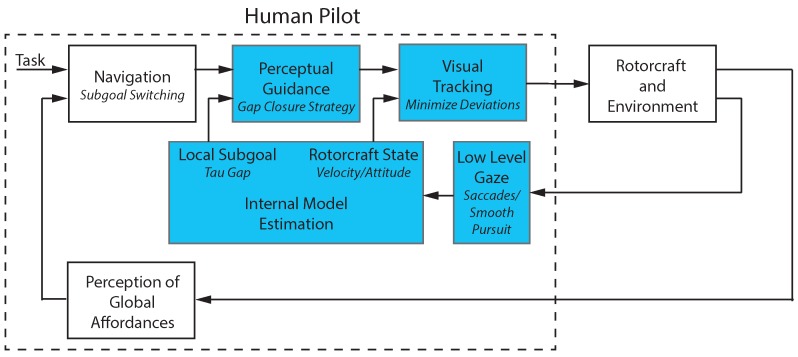
General human–machine system model for human perception and action.

The work in this paper builds on the multi-loop model of human control shown in Figure 2. The different blocks in the diagram represent the system components that comprise a human pilot’s perception and action. The model is defined as a hierarchical control system that was introduced in [5]. In Figure 2, the outer navigation loop performs a planning function that identifies the current subgoal and switches between subgoals when necessary. The navigation block operates at a higher level of abstraction. It takes as input a task definition and the location of environmental objects relevant to the task, for example the marker for a goal location, and outputs the currently active subgoal. The next loop in the hierarchy, the perceptual guidance loop, generates trajectories that will maneuver the vehicle to the specified subgoal. The perceptual guidance block takes input information about the goal location and vehicle state to generate a trajectory that will close the gap between the two. The inner loop performs a tracking and pursuit function that follows the desired trajectory while rejecting disturbances. The visual tracking block inputs the desired trajectory from the visual guidance block along with the estimated vehicle state and uses feed-forward control to generate an open loop vehicle motion. A feedback control loop minimizes the tracking error.

To perform these functions, perceptual processes are required to extract information about the vehicle state, local subgoal, and environmental affordances. Affordances are features of the environment that present an opportunity for action that are compatible with the constraints of the sensory-motor system. The perception of global affordances block in Figure 2 identifies environmental features relevant to the current task. In this work, the affordances are marks on the ground and their associated guidance actions that turn these elements into sub goals. The low-level gaze block decodes the combined eye and head motion into saccades (rapid eye movements) and smooth pursuit (eye movement tracking a moving object). The internal model estimation block captures the operator’s ability to estimate key information in their internal reference frame. The block takes the decoded saccade and smooth pursuit points to update the estimates of the vehicle and goal locations. The block outputs the estimated rotorcraft state along with the perceived gap between vehicle and goal (called the tau gap). These individual blocks and more in-depth definitions of terms are described in subsequent sections of the paper.

### 1.1. Motivation

Dynamic teleoperation in complex environments requires a human to extract task-relevant information from the optic array and generate an appropriate control response. Successful teleoperation relies on capabilities including guidance, trajectory following, feedback control, and environmental perception. Modeling the perceptual and control processes of the human operator provides the foundation necessary for a systems-based approach to the design of control and user displays used by remotely operated vehicles. When modeling the human-in-the-loop, the dynamics of the vehicle, environment, and human perception–action are tightly coupled. The dynamic response of the overall system emerges from the interplay of perception and action. The primary goal of this work is to investigate the structure of the human pilot’s perceptual and control processes and identify suitable models. The model structure builds on existing dynamic models of eye/head motion and requirements for control response during goal interception [6]. The specific control requirements are derived from the functionalities needed to support the gap closure, in particular the vehicle state and goal state that determine gap information, where the measurement update is provided by the visual gaze saccades. The parameters for the resulting model are identified using system identification. Once identified, models of the human-in-the-loop can be used to design more natural and intuitive control interfaces that tap into the innate mechanisms and therefore reduce the operator workload and allow the human and automated systems to each act in their areas of strength.

#### 1.1.1. Teleoperation Example Application

Teleoperation applications cover a broad range of domains such as exploration, surgery, inspection, search and rescue, and surveillance. Remote teleoperation applications require the operator to perform tasks based on limited information using perceptual processes that are usually structured to conform to the hardware constraints rather than natural human capabilities. Typical hardware constraints include inadequate video resolution, limited field of view, and poor depth information. Successful completion of remote tasks requires overcoming these limitations while maintaining situational awareness. This work investigates automating remote camera movement based on models of head–eye coordinated motion and augmenting the control system to assist the operator. The goal is to improve situation awareness and reduce the operator workload by augmenting the basic teleoperation configuration with aids that emulate the natural visuo-motor system.

**Figure 3:**
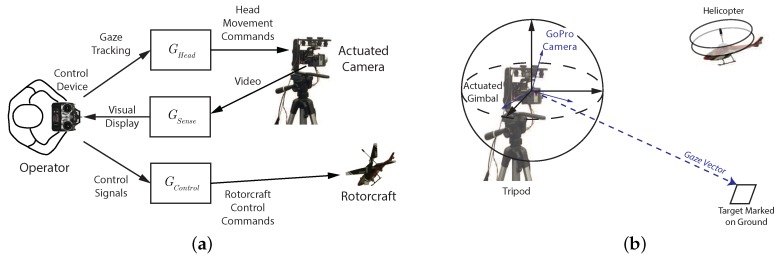
Teleoperation block diagram and physical representation. (**a**) The teleoperation configuration showing the information filters on the sensing and control capabilities of the operator. (**b**) Teleoperation camera, gimbal, vehicle, and task element.

The most common teleoperation configuration utilizes a live video feed to provide information about the remote environment as shown in Figure 3. Two key considerations that need to be addressed for the teleoperation configuration are situational awareness and pilot workload. To allow for adequate situational awareness, the camera must either have a large field of view or be controlled by the operator. During highly dynamic and interactive tasks, like operating a miniature rotorcraft or performing surgery, it is not feasible for the operator to manually adjust the camera while simultaneously performing the task. If a fixed camera with a wide field of view is used, problems can arise due to inadequate resolution in the area of interest or the need to operate outside the set field of view. The pilot workload while performing flight tasks can be high due to fast acting dynamics and the unstable nature of the vehicle. Determining the appropriate level of automation can be critical for reducing workload without impacting performance.

In teleoperation applications, the information flow is limited in both directions. Figure 3a shows the components of a teleoperation system. The information filters $G_{Head}$, $G_{Sense}$, and $G_{Control}$ represent the fact that the operator’s sensing and control capabilities are modified by the teleoperation infrastructure. On the remote side of the system, the control interceptor (like a joystick) is often the only input information available. With advances in eye tracking technology, inexpensive systems are becoming available. Eye tracking measurements can be a rich source of information that can be exploited to improve the perceptual processes as well as providing knowledge on the dynamics of the remote vehicle. For the teleoperated system shown in Figure 3b, the gaze is reproduced by a camera on a gimbal system which creates the superposition of the human head and the eye orientation. The system is driven by the gaze measurements while observing a display rather than directly observing the environment. Therefore, the gaze vector for the teleoperation system is the combination of the gimbal orientation and the tracked eye position on the teleoperation display and captures the vector going from the gimbal location to the operator’s focus of attention. Gaze provides a measurement of the human perceptual state and can be exploited to estimate the state of the controlled vehicle as well as the key environmental features of the remote system [7].

To improve teleoperation performance, models of the pilot’s perceptual and control capabilities are identified and used to automate parts of the teleoperation task. Section 6 provides details on the systems implemented along with discussion of the performance improvements.

#### 1.1.2. Human-in-the-Loop Systems

For many operating scenarios, fragile automation systems are unable to provide adequate performance. In contrast, human-in-the-loop systems demonstrate an ability to adapt to changing and complex environments: they find stability in control response; they achieve high-level goal selection and planning; and they possess the ability to perceive and process large amounts of information. During teleoperation tasks, the human operator and the automated systems provide complementary capabilities with the human able to excel at high-level reasoning, task determination, spatial mapping, and guidance, while the automated systems excel at dynamic regulation, trajectory optimization, and path following. Fitts [8] described the trade-offs of human-in-the-loop systems and characterized the complementary capabilities of humans and machines. Table 1 lists the relevant strengths of each.

### 1.2. Research Objectives

The central research question investigated in this paper is the following: how does the human operator structure their control and guidance response? The general approach is based on modeling the human–vehicle–environment interactions based on dynamic systems and controls followed by the application of system identification. Secondary questions to be answered include (i) what is the representation of the information that is visually extracted by the human; and (ii) how is the operator gaze participating in the estimation of the vehicle state and relevant task elements.

Figure 2 illustrates the general system model proposed to capture the human perception–action interrelation for teleoperated guidance and control tasks. The control theoretical view of human pilot modeling formalizes the pilot, the vehicle, and the environment as a system that observes the current state, compares this state with a desired state, and then takes action to move the current state towards the desired one. This knowledge is also relevant for the development of interactive robotic systems. In particular, the concepts relating to perception and adaptability apply to any system where the dynamics of human interaction are critical. The work in this paper focuses on modeling the system components highlighted in Figure 2. The non-highlighted blocks for the higher, navigation-based level are being investigated in separate research efforts [9,10,11]. Once models are identified, the components are implemented as part of a teleoperation system in order to alleviate the operator workload and provide a natural interaction that mimics human head motion.

This paper proposes a model structure that integrates human perception, internal state estimation, trajectory generation, and control. The objectives of this work differ from existing research by taking what have largely been conceptual ideas on action–perception and structuring them to be implemented for a real-world problem. The key contributions are (1) the novel approach for utilizing gaze as the primary sensory mechanism for measuring vehicle state and task elements; (2) the representation of the human pilot’s internal model of the vehicle state and task elements (Internal Model Representation block in Figure 2) that uses a body centric spherical reference frame corresponding to human visual perception; and (3) models of the pilot’s perceptual guidance and visual tracking processes. The proposed model was implemented to provide natural augmentations for teleoperation that simplify perception and control for the human pilot. Example applications demonstrate the benefits.

### 1.3. Paper Organization

The paper is organized as follows. Section 2 reviews related work and background. Section 3 provides a brief overview of the experimental setup. Section 4 details the human control response model with Section 5 discussing the role and contribution of the operator’s gaze motion. Section 6 presents example applications that automate the positioning of a remote camera based on the operator gaze behavior and augment the flight control system to simplify the task for the pilot. Finally, Section 7 provides conclusions.

## 2. Related Work

This paper models the perceptual and control processes of a human pilot operating in a third-person perspective. Work investigating a first-person perspective can be found in [12,13]. To begin, general approaches for modeling a human’s behavior and perception are discussed. The following sections focus on specific aspects of the larger problem such as guidance behavior, perceptual guidance, human control models, and human gaze.

### 2.1. High-Level Human Models

In the literature, cognitive models have been developed by researchers from a variety of disciplines including psychology, computer science, robotics, human–computer interaction, cognitive science, neuroscience, and human factors engineering. These models are high-level, conceptual constructions intended to cover a broad range of human behavior. These models propose high-level structures for human processing but lack many of the necessary details to implement for real-world scenarios. This section describes the key work done in relevant research areas.

#### 2.1.1. General Cognitive Models

Action regulation for complex systems was discussed by Dörner [14]. The work breaks down the process into phases including goal elaboration, hypothesis formation, prognoses, planning, monitoring, and self-reflection. Errors corresponding to each phase are described along with potential reasons for the mistakes. Albus [15] proposed a multi-scale planning model that used a hierarchical structure to model human response. In this approach, the abstraction of the representation increases with higher levels while the resolution decreases. Both Dörner and Albus provide concepts that are inherent to the model structure in Figure 2. A final relevant model was proposed in [16]. In the model, the levels in a nested hierarchy have increasing bandwidth when moving from outer to inner loops.

Pew [17] discussed the structure of human perceptual-motor performance and identified three levels of organization. The lowest level acts as a simple servomechanism that generates motor outputs to correct differences between the perceived and desired state. The next level captures the human capacity to identify and implement patterns of motion based on the predictability of task and environment. The final level considers the goal and environment to call from memory integrated patterns of movement. The structure defined by Pew is similar to the approach utilized in this work. However, this work takes the conceptual ideas and seeks to identify detailed perceptual and control models.

#### 2.1.2. Human Perception–Action

Gibson’s school of ecological psychology was the first to emphasize the agent–environment coupling [18]. The ecological approach to perception described a dependence between the operator’s control response and perception. The control response is driven by the perceived state of the vehicle and environment, while perception is largely defined by the movement resulting from control actions. Consequently, attempting to study the perception and action problem by focusing on either perception or action alone only captures part of the problem.

Gibson also coined the term “affordance” to represent features of the environment that present an opportunity for action [19]. Investigation into the perceptual aspects of affordances include work on the accuracy of affordance perception [20], relation to body dimensions [21], and affordances that account for movement capability [22]. Using affordances as part of a control strategy to guide action was discussed in [23].

Based on the ecological psychology movement, research interest in a more formal dynamics- and control-based theory of perception and action has grown. Warren proposed a simple model of behavior dynamics that describes the agent and environment using dynamical systems theory [24,25]. Warren’s approach integrates four main ideas: (i) the agent is embedded in the environment; (ii) control is based on information about the agent–environment state; (iii) control actions are specific to the current task; and (iv) behaviors result from agent–environment interactions.

Applications of this model have emphasized the role of dynamics in coordination, however, results are mostly limited to simple tasks such as balancing an object [26], bouncing a ball on a racket [27,28], intercepting a moving target [29], or walking [30,31,32]. A well-known example is the catching of a fly ball by a baseball outfielder [33].

### 2.2. Human Guidance Behavior

Guidance includes a range of dynamical interactions, starting with the vehicle or body itself, and then extending into the dynamics that encompass the entire human–machine or agent–environment [34]. When humans operate in natural environments, such as piloting in complex terrains or performing surgery, they have to learn the patterns of interaction between the environment and motion, as well as learn to extract useful visual cues. In [9], a mapping technique was introduced to study the spatial characteristics of ensembles of trajectories collected from precision interception experiments. *Interaction Patterns* (IPs), which are structural features emerging from the dynamical interactions in the agent–environment system, have been proposed as a way to formalize these concepts [9]. The IPs let a human organize their behavior in ways that mitigate the various sources of complexity. Invariants in this larger system are expected to play a central role in shaping the architecture responsible for integrating controls, perception and planning processes. These results were integrated under a hierarchic model in [5].

The concepts presented in [5] were applied for studying learning and perceptual control mechanisms. In [35], the role of constraints associated with the biological mechanisms and task structure in shaping human behavior are discussed. This perspective is used to study the formation and evolution of interaction patterns over successive trials, and shows that interaction patterns can be used as basic elements of the task environment representation. This model enables the evaluation of the learning process and assessment of the operator performance (see [11] for details). The paper also describes how interaction patterns can be considered as functional units. Segmenting and aggregating behavioral data according to the structural features found in the interactions enables detailed modeling of the underlying control mechanisms, in particular the perceptual guidance (see [13] for details). This structural perspective is applied in the present paper to the details of the gaze dynamics.

### 2.3. Perceptual Guidance

Models to explain perceptual guidance have been investigated for both animal and human guidance behavior. The most widely accepted approach, called tau theory, originates from Gibson’s ecological psychology and was proposed by Lee [36]. The central idea is that the visual and motor systems form a unified perceptuo-motor system. The system utilizes a biological variable τ that represents the time-to-contact at the current closure rate. In the simplest form $\tau =y/\dot{y}$, where *y* is the motion gap and $\dot{y}$ the rate of closure of the gap. One of the main benefits of the theory is that the τs are naturally extracted by the visual system. Another strength of this mechanism is its simplicity, which enables real-time implementation. The theory was extended to include the concept of an intrinsic action gap generated internally and how that is coupled to the physical action gap [37].

Tau theory has been verified for numerous simple control tasks in humans and animals. Examples include bird landing [38], hummingbirds docking on a feeder [39], foot landing during long jumping [40], and drivers braking [36]. More challenging examples involving the coordination between motion in multiple dimensions, as well as the incorporation of tau guides, are discussed in [37]. Recent work has utilized the theory for investigating helicopter pilot behavior [41,42,43].

### 2.4. Human Control Models

Since the middle of the last century, researchers have worked to model human control actions, especially in the case of piloting an aircraft. This section details work on characterizing the human pilot and discusses how the response can be modeled as a multi-loop system.

#### 2.4.1. Control Theoretic Models of Human Performance

Research efforts towards modeling the human as a controller first began in the 1940–1950s to study human motor performance with the first significant publication coming from Tustin in 1947 [44]. The research investigated the manual control response of an operator targeting a gun turret. The main contribution of the work was to demonstrate that a linear control law with remnant could describe the operator response. Elkind performed experiments using a wide variety of inputs constructed from a number of sine waves of different amplitude and frequency [45]. From the data, transfer functions were identified that covered a wide range of system characteristics.

One of the primary results in the field of operator performance modeling was the work of McRuer on the crossover-model [46]. It was shown that for human-in-the-loop feedback control systems, the combination of the human operator and the system dynamics can be approximated by a simple integrator with a delay system near the crossover frequency $\omega_{c}$ [47].

McRuer continued work in the area developing the quasi-linear control model [46]. Based on the cross-over model and linear control models, McRuer identified the transfer function of the human operator for a number of system dynamics types [48,49]. The work showed that the same general loop transfer function $L\left(s\right)=\omega_{c}/s$ was valid for a number of situations with the human operator adapting performance to compensate for the system dynamics. These models focus on tracking and pursuit tasks in which subjects track a given visual stimuli.

#### 2.4.2. Multi-Loop Control Analysis

In the 1960s, Krendel introduced the Successive Organization of Perception (SOP) framework [50]. The framework described a progression of human control skill that starts out as compensatory, moves to a pursuit type organization, and finishes as an open-loop response. SOP describes the human internal processes that develop as skills improve. The idea can also be seen as identifying the structural blocks necessary to capture the control response of a human pilot, namely the compensatory, tracking, and open-loop components.

**Figure 4:**
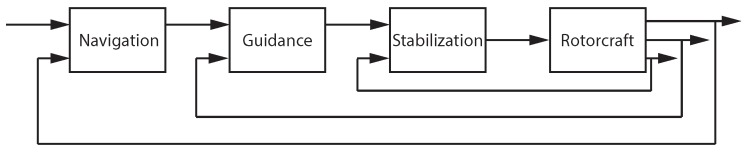
Multi-loop pilot model structure.

The multi-loop pilot model is essentially a form of embedded agent–environment model. It describes the human control behavior in terms of a nested series of control loops of increasing bandwidth and was proposed to describe pilots’ manual control [51,52,53]. The loops are organized hierarchically as shown in Figure 4 starting with the low-level attitude stabilization. Next, a guidance element generates trajectories to achieve the desired objective. Finally, the navigation block performs goal identification and directs high-level maneuvring.

Analysis of pilot performance based on an integrated pilot–vehicle multi-loop model for helicopter maneuvers was proposed by Heffley [54]. The pilot modeling work was extended into the Adaptive Pilot Model (APM) by Padfield [55]. The APM is based on the concept that the pilot converts the complex coupled pilot–vehicle system to a simple relationship between command and control output. The model utilizes a multi-loop architecture to capture the pilot response.

### 2.5. Biological Motor Control Theories

Several general theories of human motor control have been proposed. For example, Todorov in [56] investigated why redundant human motions are successful while at the same time exhibiting wide variability in trajectories. An optimal feedback control approach was proposed where variability was allowed in task-irrelevant dimensions. The present paper models the interactions of the operator’s gaze and control behavior, following the hypothesis that human remote control is conditioned by gaze dynamics and other functional constraints in particular perceptual guidance mechanisms. Another important distinction of this work relative to optimal control applications in human behavior is that it focuses on the interaction with the environment and the sensory-motor system. Optimal control has primarily focused on biomechanics, such as gait, posture, or athletic maneuvers (e.g., jumping).

### 2.6. Gaze Modeling and Classification

Human vision provides the primary source of information for humans’ everyday activities, from ordinary behaviors like reading, walking and driving [57,58], to highly specialized tasks like surgery, tele-operation and sports. Visual perception is achieved via the deployment of a foveated visual system [59]. The fovea spans a small optical angle in the visual field where high resolution information is acquired. Humans extract knowledge about the environment by actively orienting the fovea with the coordination of eye movement and head movement. This coordinated eye–head motion is called gaze. This section looks at work on modeling the eye–head motion as well as identifying the different gaze modes of operation.

#### 2.6.1. Gaze Models Based on Eye–Head Coordination

Gaze movement is the transition of visual focus in space, which involves both eye movement relative to head, and head movement relative to space [60]. That is, gaze control encompasses the entire eye–head coordination, which is attenuated by the vestibulo-ocular reflex (VOR). The eye–head coordination has been frequently investigated. Bizzi [61] proposed that the eye movement is programmed based on the planned head movement. In contrast, Guitton and Volle [60] suggested that the gaze control system can utilize all available components synergistically. More specifically, Wijayasinghe, et al. [62] pointed out that the VOR would rotate the eye backward to compensate for the forward movement of the head, minimizing the cost.

Head–eye coordination during gaze shifts has been mainly investigated in the psychophysics field. Models explaining gaze orienting to targets have been determined for both ‘within’ and ‘outside’ the occulomotor range [60,63,64]. The model describes the combined head–eye dynamics during *gaze shifts*. Two aspects of the model that are noteworthy are the independent control of the head and motor systems, i.e., head motion can be controlled as a separate system, and that initiation of eye and head movement is controlled by different gating mechanisms.

In addition to *gaze shifts*, the *smooth pursuit* eye tracking mode is also relevant for teleoperation systems. Smooth pursuit occurs when the operator is visually tracking a moving stimulus. During smooth pursuit, the eye remains focused on the moving object with the head–eye motor system coordinating the gaze motion. Lisberger proposed a closed-loop structure that generates the smooth pursuit response [65].

#### 2.6.2. Gaze Classification

Recent advancements in eye tracking systems technology have enabled the study of the mechanism of active gaze movements during diverse tasks and conditions. Three gaze patterns (fixations, saccades, and smooth pursuit) have become widely accepted and provide essential insights into gaze movement. It is only during fixations and smooth pursuits that high quality visual information is acquired. Smooth pursuits are used to update the dynamical state information needed for regulation [7]. Fixations are tightly linked in time to the evolution of the task [58]. High velocity and short duration of saccades render the visual system essentially blind, but they also reflect the economy of human attention organization [66].

Gaze classification has been realized based on the distinction in the kinematics of the three gaze patterns, i.e., by setting respective thresholds of velocity and duration range. Salvucci and Goldberg [67] focused on fixation identification and proposed a taxonomy of classification algorithms with respect to spatial and temporal characteristics, including Velocity-Threshold Identification (I-VT) and Hidden Markov Model Identification (I-HMM).

## 3. Experimental Setup

This section briefly describes an integrated research environment specifically developed to exercise and investigate guidance and control capabilities under human control, autonomous control, and augmented control modalities. The lab facility is designed to implement tasks that emphasize agent–environment interactions. The overall goal is to characterize these interactions and to apply the gained knowledge to determine models of the underlying perceptual and control processes. The approach is to combine data-driven methods with theoretical investigation through the application of formal modeling and analysis techniques from dynamics and controls. The facility uses miniature rotorcraft as test vehicles with a Vicon motion tracking system and SensoMotoric gaze tracking system.

### 3.1. Experimental Infrastructure

**Figure 5:**
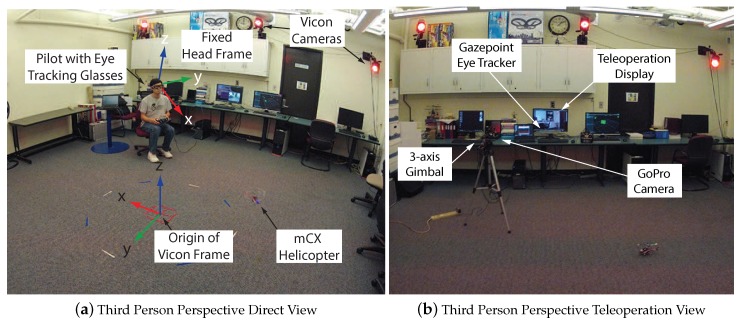
Overview pictures showing the lab setup for flight experiments with the pilot (**a**) directly observing the rotorcraft (**b**) operating in a teleoperation configuration.

The research agenda requires being able to run experiments with actual hardware components that combine the effects of vehicle dynamics, environmental sensing, and measuring a human’s perception and action. The lab facility was set up to use small-scale rotorcraft UAVs due to their maneuverability and compact sizes. Figure 5a shows an overview of the lab infrastructure where the pilot operates in a “third-person” perspective, i.e., the operator views both the vehicle and task from outside. A camera mounted on an actuated gimbal provides a teleoperation setup with a “third-person” perspective as seen in Figure 5b.

For experiments with human subjects, measurement of the operator control inputs, head pose, visual gaze, and field of view video are collected along the vehicle motion to provide data for the investigation of the control and perceptual functions. The experimental procedure and methods rely on the collection of ensembles of trajectories. Ensembles include data from multiple experimental trials that cover the task space of interest. Using ensembles allows sampling of the human behavior over larger domains and thus provides a means to extract information about the larger strategies used for planning. For more details on the lab hardware and software systems, see [68]. The gaze registration and classification approach is discussed in [69].

### 3.2. Experimental Tasks

This section describes the experimental tasks investigated in this work. The tasks each emphasize different aspects of a human’s interaction with the test vehicle and an environment.

#### 3.2.1. Hovering

**Figure 6:**
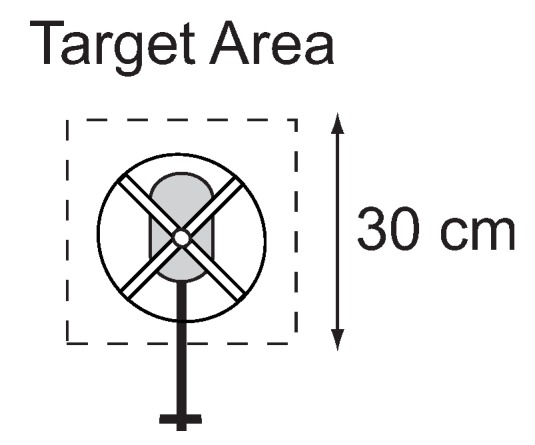
Proposed experiment for hovering.

The experiment in Figure 6 involves the human pilot maintaining a stationary hover over a target marked on the ground. For a given length of time, the pilot will minimize the longitudinal and lateral position error to achieve a stable hover. The experiment lasted 60 s for each trial.

This task formulation isolates the perceptual mechanism used by the pilot during simple regulation. The hypothesis is that the human pilot focuses attention on the helicopter and uses rapid eye motions (saccades) between the vehicle and the target to maintain a stable hover.

#### 3.2.2. Target Interception

**Figure 7:**
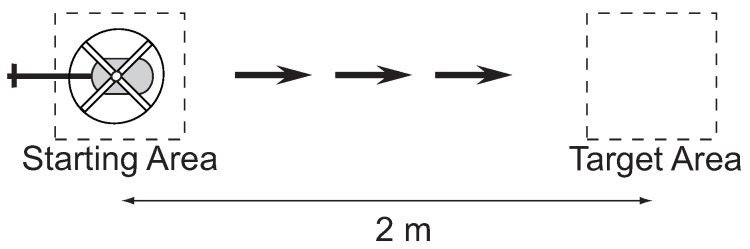
Proposed experiment for intercepting a designated goal location.

The experiment in Figure 7 involves the human pilot flying directly to a target. This task exercises the human’s low-level tracking control and the perceptual process for extracting the feedback control signal. The pilot was instructed to start from a stationary hover over the starting area and then maneuver the vehicle to the target area at one of three speeds: slow, medium, and fast. The experiment was conducted 10 times at each of the different speeds. For each speed, the operator is instructed to keep the velocity as consistent as possible between the 10 runs.

The purpose of the task is to isolate the basic building blocks for modeling human perception–action. These blocks include the structure of the single-loop feedback control action, the representation of the feedback error signal, and how the reference trajectory is generated (tau guide). The significance of the task is that it isolates the basic blocks that are necessary for understanding more complex behaviors.

The first hypothesis is that tau theory, developed by Lee [36], provides a method for reference generation that is applied through a feed-forward element to generate an open-loop response that maneuvers the vehicle from the start to the target. In Tau theory, a human utilizes a biological variable τ that represents the time-to-contact at the current closure rate, for example the time-to-impact when breaking a car. The basic form is $\tau =y/\dot{y}$, where *y* is the motion gap and $\dot{y}$ is the rate of closure of the gap. The human manipulates the control (break pedal) to maintain a $\dot{\tau }$ that ensures stopping before collision. A $\dot{\tau }>-0.5$ will cause the vehicle to stop short of the obstacle, while $\dot{\tau }<-0.5$ will result in the vehicle colliding with the obstacle (a $\dot{\tau }=-0.5$ will stop right at the obstacle). The second hypothesis is that existing research on human pilot modeling, based on linear control, can provide a reasonable algorithm for the model of the human feedback control action. Works by McRuer [48] and Hess [70] have shown that a linear model structure can capture the human control characteristics. The third hypothesis is that the gaze provides information on the reference trajectory.

### 3.3. Characterization Methods

When evaluating different control, sensing, and display systems, it is necessary to be able to measure changes in the performance and operator workload for a defined set of tasks. This section describes the metrics utilized. The metrics can be broken into three categories: speed, accuracy, and operator workload. The definition of these metrics is given below.

#### 3.3.1. Speed

For the target interception experiments, the speed is calculated based on the time *T* to maneuver from the starting position to the target. Since the distance is constant, the velocity is v=2/T m/s. Speed measurement is not relevant for the hover task since the objective is to keep the helicopter stationary.

#### 3.3.2. Accuracy

Performance can be evaluated based on the speed–accuracy trade-off. In 1954, Fitts published research on the relationship between speed, movement distance, and accuracy. The approach utilized a “Fitts Task” where an object was moved along a linear path between two locations [71]. The relationship is given by

(1) $$Movement\;Time=a+b\log_{2}\left(\frac{2A}{W}\right)$$

where *A* is the distance between locations, *W* is the target width, and the linear relationship (constants *a* and *b*) is empirically identified. A revised relationship was proposed by Schmidt [72]. Schmidt found that for tasks requiring a single-aimed movement, a linear relationship existed between the movement speed and the effective target width. The revised relationship is

(2) We=a+b(AAMovementTime)MovementTime)

where $W_{e}$ is the effective target width. To calculate $W_{e}$, the within-trial error was measured to find the standard deviation or “spread” around the target locations. The effective width, $W_{e}$, was defined as the width necessary to capture 96% of the identified distribution.

#### 3.3.3. Workload

An important measure for assessing a helicopter system for a task is how much workload is required by the operator to successfully perform the task. The challenge in objectively and quantitatively addressing this question is that both task and human control include measurable quantities, such as control signal variation as well as subjective measures of the task difficulty [73].

The workload metric is a measure defined by the attention functional in [74]. Brockett defined an attention metric based on the control signal *u*, state *x*, and time *t*. The original metric had two terms ${\left(∥\frac{\partial u_{i}}{\partial t}∥\right)}^{2}$ and ${\left(∥\frac{\partial u_{i}}{\partial x_{i}}∥\right)}^{2}$. In this work, we focus on the first term which looks at how the control signal changes versus time. The functional is discretized with samples *i* and the resulting metric is defined by:

(3) $$\eta =\frac{\sum_{i}{\left(∥\frac{\partial u_{i}}{\partial t}∥\right)}^{2}}{T}.$$

The discrete approximation of the attention functional is intended to be a measure of how much effort the operator needs to supply in order to complete a task. In general, the amount of effort is roughly proportional to the magnitude and frequency of the control adjustments that the operator needs to make. When executing a task where the control inputs are held relatively constant (such as a stable hover), the attention functional would be small. A task that requires significant control (such as navigating a slalom course) would have a much larger attention measurement.

### 3.4. Test Pilots

Six undergraduate students from the University of Minnesota performed the flight experiments in Section 3.2. All subjects gave their informed consent for inclusion before they participated in the study. The study was conducted in accordance with the Declaration of Helsinki, and the protocol was approved by the Ethics Committee of the University of Minnesota.

The pilots were recruited from the aerospace department and had past experience operating RC helicopters. The test pilots demonstrated a range of skill levels. Two pilots possessed significant experience and were able to complete tasks quickly and accurately. Another two pilots had some experience operating miniature rotorcraft but performed with less accuracy than the highly skilled operators. The last two pilots in general displayed the least accuracy. The modeling work in subsequent sections was based on the test data from the highly skilled pilots.

Flight tests were conducted in one-hour sessions that occurred weekly. At the start of each session, a calibration procedure was required for the eye tracking system. The pilots were then allowed to practice the task until they felt comfortable. To maintain consistency between experiments, the pilots were seated at the same location for all test flights.

## 4. Model of Pilot Control

Human control requires a number of processes and mechanisms acting in concert to achieve the level of performance seen in skilled individuals. Pew identified three levels of control organization [17]. The most basic level of control generated by a human pilot is that of a simple servomechanism where motor outputs act to correct the error between the perceived and desired states. This basic control element can be represented by concepts from the theory of feedback control and provides a basis for all higher levels of control organization. The next level of control incorporates the coherence and predictability of the task and environment. The human pilot generates a desired trajectory based on patterns of behavior learned from past experience. The last level of human control draws cues from the environment that identify a goal, which then recalls from memory an integrated motion model that can achieve the desired result. This section models the two lower levels of the human pilot’s control organization for simple flight tasks.

### 4.1. Visual Tracking: Linear Control

**Figure 8:**
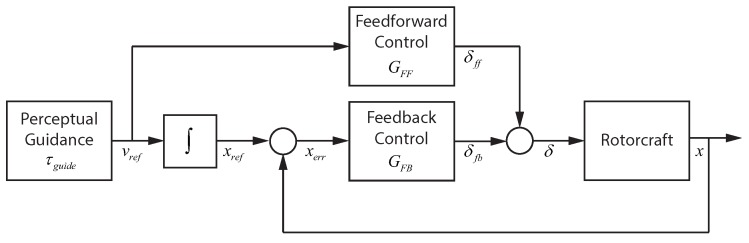
Structure of the visual tracking loop.

To operate a vehicle along straight trajectories, the visual tracking component can be modeled using dynamic linear elements. Figure 8 gives a detailed breakdown of how the visual tracking component can be implemented. The visual tracking operates in the dimension of the task motion with the single output mapped onto the lateral and longitudinal control outputs. In the figure, the perceptual guidance block generates a reference velocity, which passes through a feed-forward element to generate a control signal $\delta_{fb}$ that drives the vehicle velocity to match $V_{ref}$. In parallel, the reference velocity is integrated and compared with the actual position to generate a positional error $x_{err}$. The positional error provides the input for the feedback part of the visual tracking system. The feedback element generates an error correcting control signal $\delta_{fb}$. The control signals $\delta_{fb}$ and $\delta_{ff}$ are added to obtain the output of the visual tracking block δ.

#### 4.1.1. Feed-Forward Control

The feed-forward control element takes a velocity reference and outputs a control signal that produces the desired velocity in the helicopter. A dynamic model of the miniature rotorcraft used in the flight experiments was identified using system identification techniques. A simplified version of the model from the longitudinal control signal $\delta_{lon}$ to the vehicle’s forward velocity *u* is given in Equation (4).

(4) $$G_{\delta_{lon}u}=\frac{2.802}{s+2.029}$$

Inverting the simplified model of $G_{\delta_{lon}u}$ gives the feed-forward dynamics that map a velocity reference $V_{ref}$ to control signal $\delta_{ff}$ that will drive the vehicle to the desired velocity. Equation (5) gives the dynamic model for the feed-forward block $G_{FF}$.

(5) $$G_{FF}=0.724+0.357s$$

It should be noted that $G_{FF}$ is outside the feedback loop and potentially sensitive to high-frequency noise. The functionality of the feed-forward path should mitigate high-frequency issues as the velocity reference entering $G_{FF}$ represents a learned operator response that is generated for a specific task. The response operates in open-loop and at lower frequencies (if necessary, a low-pass filter could be added to limit high-frequency noise).

#### 4.1.2. Feedback Control

Based on the Adaptive Pilot Model described in [53], the pilot control model takes the form of Equation (6). Estimation of the pilot control parameters for the feedback element can be accomplished using frequency domain estimation methods [75].

(6) $$Y_{P}=K_{P}+K_{D}s$$

**Figure 9:**
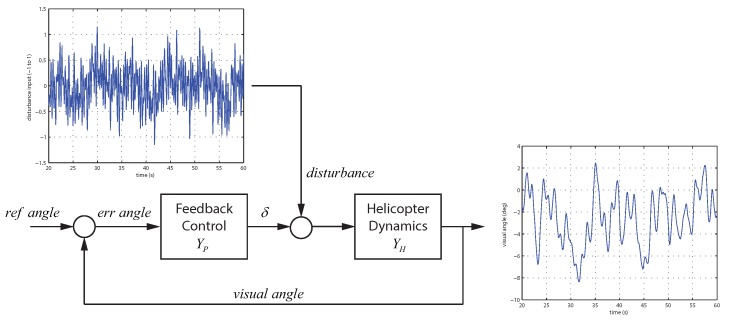
For the hover task, a known disturbance is injected into the control signal. The control parameters for $Y_{P}$ are then identified.

To estimate the control parameters $K_{P}$ and $K_{D}$, a forcing function is applied as a disturbance to the control signal as shown in Figure 9. The forcing function applied is the sum of multiple sine waves that provide a rich excitation for the system [76,77]. The multisine equation $f_{d}$ is defined by Equation (7) with the parameters for $\omega_{d}$, $A_{d}$, and $\phi_{d}$ covering the operational range.

(7) $$f_{d}\left(t\right)=\sum_{i=1}^{10}A_{d}\left(i\right)\sin(\omega_{d}\left(i\right)t+\phi_{d}\left(i\right)$$

**Figure 10:**
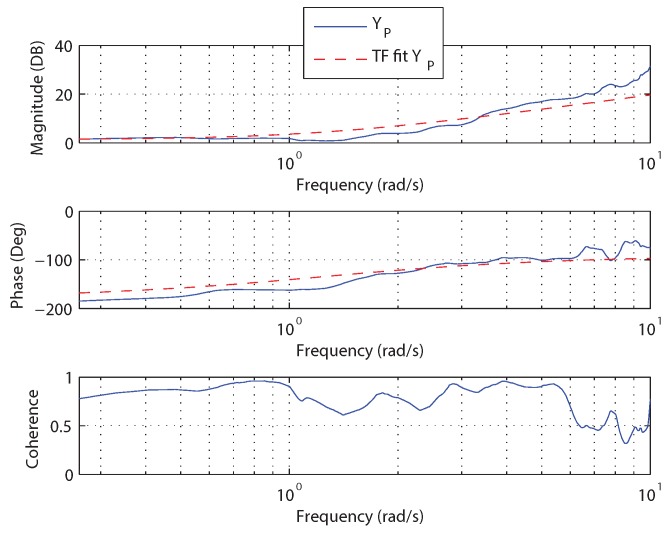
The control parameters are then identified using system identification techniques.

Data was collected for the hover task from Section 3.2.1. While the pilot performs the hover task, the forcing function is injected into the control input causing continuous displacement of the vehicle. This requires the pilot to provide control actions to return the vehicle to the desired hover position. For the hover task, the reference angle from Figure 9 is constant, resulting in the error angle being directly related to the visual angle. In this case, measurements are available for the input (errangle) and output (δ) of the feedback control block $Y_{P}$. The NAVFIT function of CIFER was used to identify the parameters of the feedback control block [78]. Figure 10 shows the transfer function fit for $Y_{P}$ along with the nonparametric frequency response extracted from the experimental data.

The transfer function fit in Figure 10 has a high coherence in the frequency range of interest (below 10 rad/s) and provides a reasonable match to the experimental data. The parameters identified for $Y_{P}$ can be found in Equation (8) with Equation (9) showing $G_{FB}$.

(8) $$K_{P}=-1.19,K_{D}=-0.94$$

(9) $$G_{FB}=-1.19-0.94s$$

In [55], it was shown that if the pilot–vehicle short-term attitude dynamics (stabilizing attitude) are assumed to follow the crossover model [46], the dynamics of the free response to a displacement for the complete pilot–vehicle system can be reduced to a second-order form with natural frequency $\omega_{n}=\sqrt{gK_{P}}$ and damping ratio ζ=gKDgKD2ωn2ωn. Factoring in the gain from the control signal δ to the attitude angle at the approximate crossover frequency ($\omega_{c}=1.0$ rad/s) gives a natural frequency and damping ratio of 1.39 and 0.55 respectively.

### 4.2. Perceptual Guidance

Research on sensory guidance has demonstrated that guidance performance can be described using relatively basic principles. The main approach, called tau theory [36], utilizes a simple variable tau. Tau is defined as the time-to-closure of a gap at the current gap closure rate. Gaps can also be closed using intrinsic guides, called tau guides, that are internally generated mental models of the desired motion [37]. When utilizing these tau guides, the externally perceived gap is coupled with the internal guide. The form of the tau guide depends on the type of motion with examples being constant acceleration, constant deceleration, or acceleration–deceleration maneuvers. Perceptual guidance can be implemented to provide the reference trajectory for a given task based on the appropriate form of a tau guide. For the step task, an acceleration–deceleration tau guide is required. The form of the tau guide $\tau_{g}$ is given in Equation (10).

(10) $$\tau_{g}=0.5\left(t-\frac{T^{2}}{t}\right)$$

The tau guide is converted into a reference velocity ($v_{ref}$) by Equations (11) and (12) which are based on the tau coupling principle and the definition of the tau variable.

(11) $$\tau_{x}=k\tau_{g}$$

(12) $$v_{ref}=\frac{x}{\tau_{x}}$$

**Figure 11:**
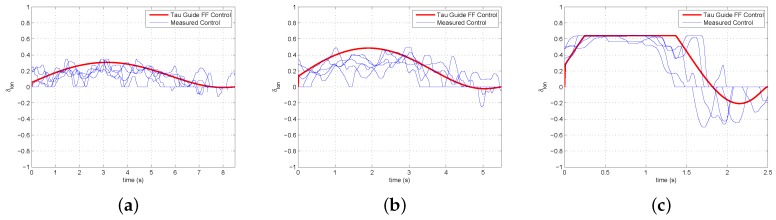
Augmented flight control provides a feed-forward control signal to partially automate the step task. A tau guide creates a reference velocity that is converted to a helicopter control signal. The subfigures show the comparison of the generated signal against a set of control human controls. (**a**) Control for Step Task at Slow Speed; (**b**) Control for Step Task at Medium Speed; (**c**) Control for Step Task at Fast Speed.

Experimental results for the step task at slow, medium, and fast speeds show that the human control response can be approximated using a tau guide. Figure 11 shows the correspondence between control signals generated using a tau guide and the actual controls from the human pilot. The value of *k* was 0.3 and the *T* values (time to complete the task) for the slow, medium, and fast speeds were 8.5 s, 5.5 s, and 2.5 s respectively. The control signal for the fast speed plateaus due to joystick limits on the maximum control action.

## 5. Gaze for Guidance and Control

The goal of the work presented in this section is to determine and model the pilot’s visual perception during precision remote-control (RC) operation of a miniature rotorcraft. For a given task, the control problem involves processing visual input and transforming the result into commands to the relevant musculature. Some of the key questions include the following: What type of information is required for the different control modalities? And how is this information extracted and used as part of the perceptual control mechanisms? To answer these questions, an approach based on the analysis of the closed-loop, operator–agent–environment interactions within a control theoretic framework is used. Figure 5a provides an overview of the experimental setup. The experiments in this work were conducted using a “third person” modality. Based on helicopter dynamics, gaze patterns, and control inputs provided by the facility, the specific goal is to identify how the gaze is integrated with human control actions.

### 5.1. Gaze Processing

The eye tracking system generates a gaze vector in terms of the operator’s head orientation. To fully utilize the information, the gaze vector needs to be registered in a common reference frame and the gaze classified into its constituent modes. This section describes the registration and classification procedures.

#### 5.1.1. Registration of Gaze and Motion Tracking Measurements

**Figure 12:**
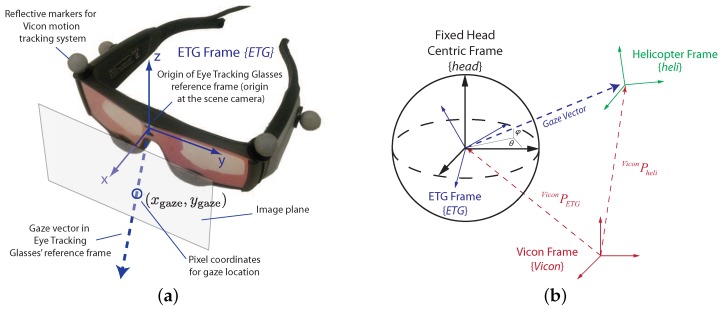
Reference Frames for Generating 3D Gaze Location. (**a**) Eye Tracking Glasses Reference Frame. (**b**) Spherical Reference Frame.

Understanding the human perceptual processes supporting guidance and control capabilities requires linking visual gaze with the vehicle, task and environment elements. The eye tracking glasses shown in Figure 12a provide a gaze vector relative to the pose of the pilot’s head. Therefore, a registration procedure is required to put the pilot’s head, helicopter, and environmental features in a common reference frame.

The determination of a reference frame for gaze is crucial for gaze classification. For instance, a 3D inertial Vicon frame is able to provide information about where human pilots are focusing, but the transition of the gaze point in this frame cannot reflect the magnitude of gaze movement. For instance, two stars are far in space but close to each other from visual perspective.

The gaze should be transformed into a reference frame appropriate for use by human decision making and motor control systems. A spherical head centric coordinate frame is proposed in [79] to describe the visual receptive field of flies and can be extended to humans. It is used in this paper and represents gaze as azimuth (θ) and elevation (ϕ) angles, as shown in Figure 12b. More in-depth details on the methods used for gaze registration can be found in [69].

#### 5.1.2. Gaze Classification

Gaze is the coordinated motion of eye and body, in which this related action is predominantly performed by the head. Fixating a stationary object while turning the head can have similar eye movement as pursuing a moving object while holding the head still. Therefore, the measurement of both head and eye motion is required to classify gaze.

Basic eye movement is comprised of three components: saccades, fixations and smooth pursuits, each having distinct kinematic characteristics. Saccades are the fast eye movements of small durations used to redirect the eye to a new location [80]. Fixations take place when the gaze is stabilized on typically stationary points [81]; their duration spans longer time intervals. Smooth pursuits are eye movements when the gaze follows moving visual stimuli [82].

These three basic eye movements can be classified according to their kinematic characteristics, more specifically, by setting the respective thresholds of velocity and time duration. For instance, the lower saccade speed limit for amplitudes of 5${}^{\circ }$, 10${}^{\circ }$, 20${}^{\circ }$, and 30${}^{\circ }$ were determined to be 145${}^{\circ }$, 196${}^{\circ }$, 213${}^{\circ }$, and 227${}^{\circ }$ per second. These characteristics were obtained by analyzing factors such as abduction, centering, eccentric, and across-the-center refixations [83].

### 5.2. Experiments

**Figure 13:**
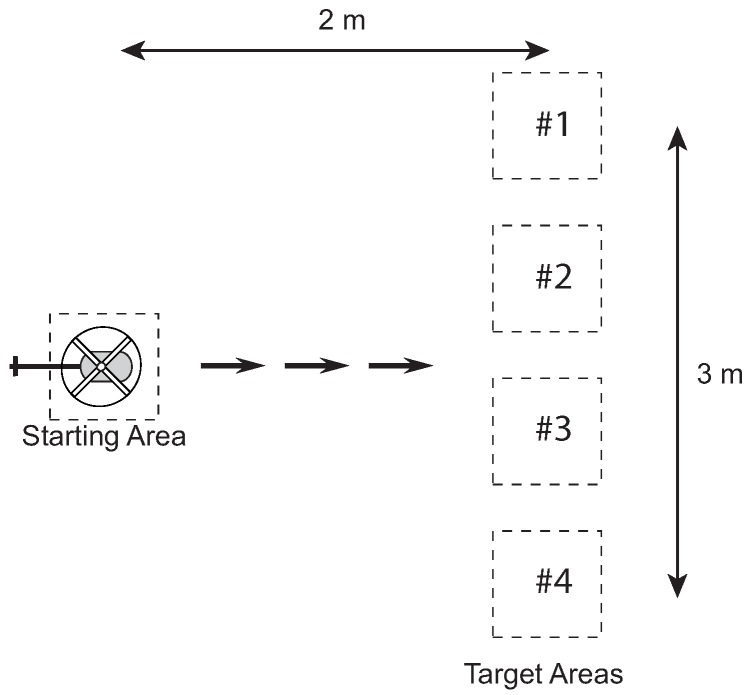
Experimental step task used to investigate the low-level control and guidance functions.

Experiments were conducted to investigate the pilots’ control capabilities. For stabilization, a hover task was used, and for goal interception, a step task. Descriptions of the hover task can be found in Section 3.2.1. Figure 13 shows an illustration of the step task used that is a variation of the one given in Section 3.2.2. Each test flight, the pilot was sitting about 2 m behind the center of the task space and was required to remain stationary during the task. Each task was performed by 4 student test pilots with skill levels varying from moderate to highly skilled. Before each session, the pilot was allowed to practice briefly before beginning a trial. The analysis in the following sections shows results for one pilot.

#### 5.2.1. Stabilization—Hover Task

In the hover task (Figure 6), the subjects were instructed to maintain the helicopter within a square marked on the floor for 60 s. In addition, the objective was to achieve the most steady hover, i.e., to minimize the velocity fluctuations. Furthermore, they were asked to keep the helicopter facing away from their body.

**Figure 14:**
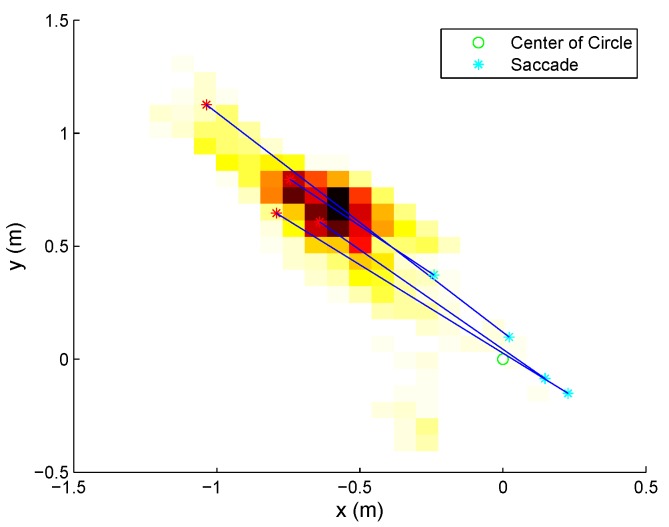
Gaze decomposition for the hover task within a small boundary area. The gaze is shown in the task space with smooth pursuit points shown as a density plot and saccades as gaze steps.

Figure 14 shows the gaze decomposed into a density plot of the smooth pursuit points and step changes for saccades for a small hover area (0.25 m in diameter). The gaze consisted of primarily smooth pursuit points with a small number of saccades to the center of the constraint area (marked on the ground).

#### 5.2.2. Interception—Step Task

The step task (Figure 13) allows isolating the processes of the perception–action loop along a single dimension. The pilots were instructed to start from a stable hover over the starting area and then perform an acceleration/deceleration maneuver ending in a stable hover over one of four target areas. The latter was specified randomly at the initiation of each trial. Random goal specification was implemented to reduce the effects of accommodation.

**Figure 15:**
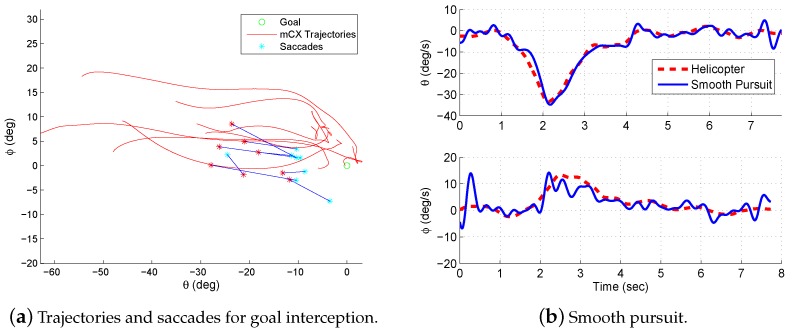
Step task results: (**a**) Saccades during step experiments (multiple trials), and (**b**) gaze pursuit mode and rotorcraft velocities in the head frame.

Multiple experimental trials were conducted. Figure 15b shows the gaze and helicopter velocities for one trial. During most of the experimental flight time, the pilot’s gaze operated in pursuit mode and tracked the helicopter closely. The gaze velocity during pursuit follows the velocity profile of the helicopter trajectory and suggests that the gaze can provide the measurement of the velocity used for feedback control.

In addition, saccades take place systematically as the helicopter approaches the goal. Figure 15a shows the saccades from multiple step trials. The data has been transformed into a reference frame with the goal at the origin and the starting locations along the negative θ (azimuth) axis. In the transformed frame, the rotorcraft trajectory moves from left to right during a trial. The saccades provide a measure of the distance remaining to the goal location. This is consistent with what is expected from tau theory. Specifically, the saccades are measuring the gap that is being closed for the task.

### 5.3. Models

The experimental results provide evidence that during remote operation, the gaze, helicopter dynamics, and control are tightly coupled. The next step is to determine models that describe the gaze dynamics’ role as part of the helicopter control mechanisms.

#### 5.3.1. Gaze Modalities Summary

The decomposition of the gaze trajectory into smooth pursuits and saccades reveals two primary patterns. First, for stabilization and tracking, the pilot needs information about the helicopter pose and velocity. Smooth pursuit gaze trajectories follow the helicopter trajectories. The gaze velocities during smooth pursuit match closely with the helicopter velocities (see Figure 15b). This information can be used for closing the velocity loop in Figure 2. Second, guidance control is clearly mediated through saccades. Figure 14 shows that the saccades move between the gaze pursuit trajectory and the target location. The saccades measure the gap (from tau theory) that needs to be closed or maintained for the task, thus providing feedback at the guidance control level.

In general tasks, the information necessary to close the guidance and control loops would be available from the scene’s visual content, the peripheral vision and the information used for the active control of gaze. During typical operation, the lower-level control modes require precise, high-bandwidth information; therefore, the assumption is that the peripheral vision plays a secondary role. This is supported by the high correlation between helicopter control behavior and the gaze pattern. For guidance tasks in unstructured environments, we would expect that peripheral vision plays a more significant role since more global information about the environment and task elements must be acquired. This aspect is beyond the scope of this section.

#### 5.3.2. Pursuit Model

The low-velocity, smooth pursuit mode is primarily operating from visible visual cues within the high-resolution region. To analyze the interaction between gaze and control during smooth pursuit, the transfer function between the gaze and helicopter velocity was identified from frequency responses extracted from input and output data. The input *x*, which is the stimulus for pursuit is the helicopter velocity, and the output *y* is the gaze velocity. Frequency response and coherence estimates are computed from

$$\begin{array}{c} T_{xy}\left(f\right)=\frac{P_{yx}}{P_{xx}};\;\gamma_{xy}=\frac{|P_{yx}^{2}|}{|P_{xx}P_{yy}|} \end{array}$$

where $P_{yx}$ is the cross- and $P_{xx}$ the auto-power spectral densities. At low frequencies (<1 Hz), the gain is one and the phase is close to zero, indicating that gaze provides a near perfect velocity measurement. The associated coherence $\gamma_{xy}$ remains large and confirms a linear input–output relationship.

#### 5.3.3. Saccade Model

High-velocity saccade motion measures distances to features in the visual environment beyond the currently visible area. Saccades, therefore, provide measurements that are needed to guide motion including the tau gaps. The motion gap can then be used to generate a velocity reference.

**Figure 16:**
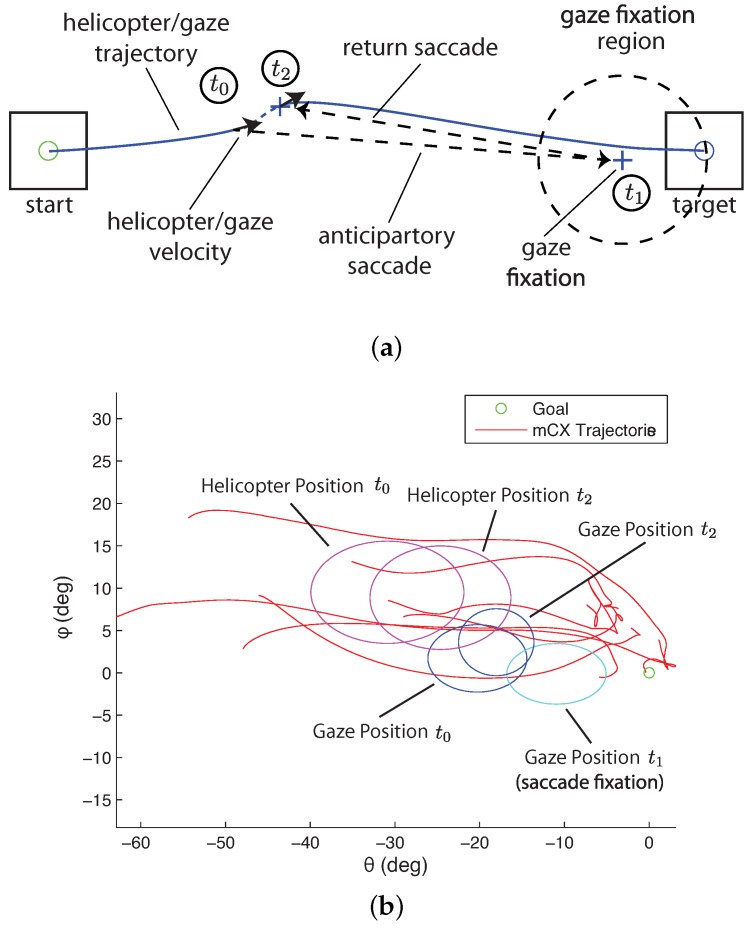
Description of the saccade dynamics during the step task. The ellipsoids are based on mean values for the position variables along with the 1σ error bounds for the step task. (**a**) Key variables involved in saccades to a target; (**b**) Statistics extracted from the step task.

The saccade mode generates information about the tau gap. The key variables for generating the tau gap information are shown in Figure 16a. The figure shows the three-stage sequence starting with smooth pursuit followed by the saccade to a fixation point ($t_{1}$) near the desired goal location and concluding with another saccade returning to the helicopter ($t_{2}$). This sequence may be initiated multiple times. For a trained pilot moving to a previously visited target, saccades may not be utilized. Figure 16b shows the position distributions during the step task in Figure 15a based on mean and standard deviation for the three times $t_{0}-t_{2}$. It is interesting to note that the saccades stop short of the goal by about 10 deg, which is enough to bring the goal within range of the central eye field.

**Figure 17:**
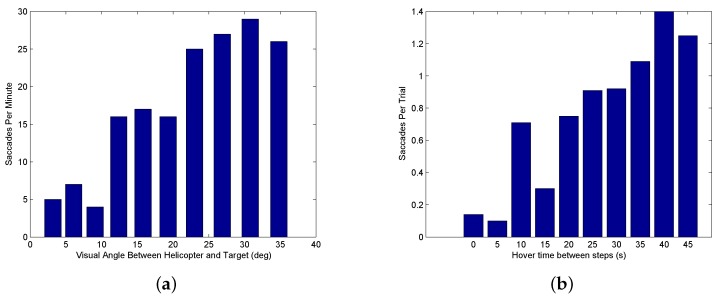
Saccade trigger factors. (**a**) The saccade frequency during hover experiments for different heights (visual angle between the helicopter and target on the ground). (**b**) The saccade frequency during step experiments for different lengths of hover time over the starting position (gives increasing lengths of time since the target location was last observed as part of the task execution).

Two factors are critical for determining when a saccade is triggered. The first is the size of the visual angle between the current gaze focus of attention and a task element. When the high visual acuity area of the gaze is close to a task element, visual information is captured about the element’s position. As the gaze focus moves farther away, information about the task element becomes uncertain and eventually triggers a saccade. The question to be answered is how close does the gaze focus need to be to capture information on the task element. Based on the anatomy of the human eye, the fovea has by far the highest visual acuity and accounts for 5 degrees of the visual field, the parafovea around 8 degrees, and the perifovea 18 degrees. Figure 17a shows a distribution of the saccades generated during a hover task with different visual angles between the vehicle and target on the ground. The visual angles are achieved by hovering at different heights. The figure shows that for 10 degrees (the fovea and parafovea regions) or less, few saccades are generated, indicating that sufficient information on the target is available and saccades are infrequent. For gaze angles from 10–20 degrees (the perifovea region), the frequency of saccades increases. Beyond 20 degrees, the human pilot is continually generating saccades to measure the target position relative to the vehicle.

The second factor in triggering a saccade is the time since a task element was last observed. According to work done by Brown [84] and numerous others since, working memory decays after around 15 s. The retention interval decreases as more items of information need to be remembered [85]. To test how long a pilot can keep track of a distant target location, a step experiment was conducted with the pilot hovering over the starting location for differing lengths of time before initiating the maneuver. As the hovering time increases, the chance for a saccade to be triggered increases. Figure 17b shows the saccade frequency for increasing time durations since the target was last observed. In the figure, the number of saccades per trial is small (less than 0.2 per trial) when there is little delay, increases to 0.7–1.0 for a 20–30 s delay, and is over 1.0 for higher delays. This indicates that for larger delays enough uncertainty has accumulated since the pilot last observed the target that a saccade is triggered.

### 5.4. Integrated Gaze and Control Model

**Figure 18:**
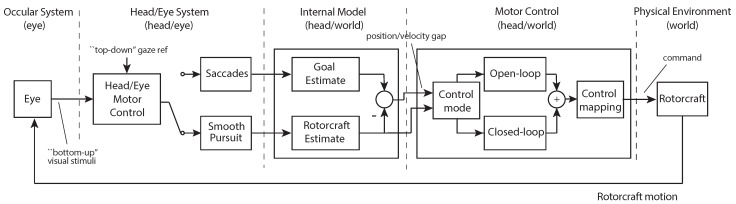
Block diagram of a notional model of the gaze control and helicopter control integration.

The block diagram in Figure 18 describes a notional model of the primary gaze and control functions and their integration based on the teleoperation experiments. To visually track an object such as a helicopter, coordinated eye and head movements must be generated (‘Head Eye System’). In smooth pursuit, the gaze keeps the visual stimuli guiding the pursuit near the center of the eye field where the eye’s resolution is highest. For goal interception, the saccades provide anticipatory information about the goal location. In Figure 18, the input to the eye block is the visual scene with the output the 2-dimensional pixel location of the eye focus in the observed image that is captured by the eye tracking system. The head/eye motor control block transforms the pixel location into the head reference frame and combines with head motion to generate a gaze vector represented by the azimuth and elevation. The saccades and smooth pursuit blocks decode the gaze vector to determine the gaze mode of operation. The goal estimate and rotorcraft estimate blocks take the saccade location and current smooth pursuit location to estimate the azimuth and elevation locations of the goal and vehicle in the spherical coordinate system (see Section 5.5 for details). Finally, the goal and vehicle location estimates are utilized by the motor control section to generate the lateral and longitudinal control signals sent to the rotorcraft (see Section 4.1).

During visual tracking and guidance, pose and velocity measurements needed to control the helicopter are derived from the motor control signal driving the head/eye system. This information is first integrated within an ‘Internal Model’ that simultaneously estimates the goal and rotorcraft state (position and velocity). This information is then used to generate a control action (via the ‘Motor Control’ system) utilizing both open-loop and closed-loop strategies. The control output can be mapped into the helicopter frame using different strategies to overcome the lack of depth information, for example assuming a constant height. For the goal interception, the tau gap extracted from the saccade information provides anticipatory information. Under conditions, entire segments of the trajectory can be generated and implemented in open-loop. Finally, as highlighted in the block diagram, the different components operate within different reference systems. In some cases, such as in the ‘Internal Model’ and ‘Motor Control’, two coordinate systems are most likely used in parallel.

### 5.5. Estimation of Vehicle and Goal States

**Figure 19:**
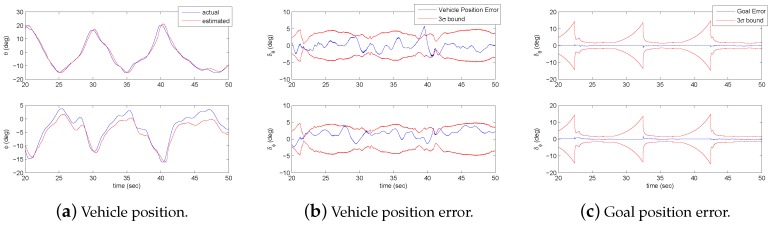
Results for the vehicle and goal position estimation using the gaze data as measurement.

The ‘Internal Model’ in Figure 18 can be described using standard state estimation techniques. The key information that needs to be estimated is the vehicle state and the position of the current task elements. A simple approach for estimating these values is achieved using a constant velocity Kalman filter designed to estimate the vehicle position and velocity. Additional states are added to the estimator to track the position of the target locations. Measurement updates are generated by the visual system. During smooth pursuit, the gaze location provides adequate information for tracking the vehicle as seen in Figure 19a. To estimate the goal position, the visual system identifies the θ and ϕ angles of the target location in the head reference frame. The distance of the visual features from the center of the fovea determines the measurement covariance. This results in high accuracy measurements when the gaze is focused on an object and low accuracy at approximately 15 deg away from the center of vision. The estimation errors along with 3σ bounds are shown in Figure 19b,c for the vehicle position and one of the goal locations. As discussed earlier, information about environmental features grows more uncertain the longer the time since the feature has been observed. To capture this characteristic, a forgetting factor λ is incorporated into the estimator and causes the error bounds for the goal position to gradually increase during the transition between locations unless a saccade to the goal occurs. Once the vehicle is near the goal position, the error bounds decrease since the goal is close enough to the high acuity visual area to provide update information.

The EKF time update equations, for a task of flying between two positions marked on the floor, are of the form given in (13). The states *x* are the azimuth (θ) and elevation (ϕ) angles for the helicopter and goal. The Jacobian calculations for the state transition matrix and the matrix characterizing model uncertainty are given by Φ and *Q*. The update equations for the state and covariance estimate *P* are denoted by the last two equations.

(13) x=[θheli,θ˙heli,ϕheli,ϕ˙heli,θgoal,ϕgoal]Φ=1dt0000010000001dt00000100000010000001Q=dt3dt333dt2dt2220000dt2dt222dt000000dt3dt333dt2dt2220000dt2dt222dt2dt2220000000.010000000.01x^k+1|k=Φx^k|kPk+1|k=λΦPk|kΦT+Q

The EKF measurement update equations are given in (14). The observation vector $\hat{z}$, observation covariance matrix *H*, measurement covariance matrix *R*, Kalman gain *K*, and updates for the state vector and state covariance matrix are given by:

(14) $$\begin{array}{c} \;\hat{z}=H{\hat{x}}_{k+1|k}\\ \;H=\left[\begin{array}{cccccc} 1 & 0 & 0 & 0 & 0 & 0\\ 0 & 1 & 0 & 0 & 0 & 0 \end{array}\right]\\ \;R_{k}=\left[\begin{array}{cc} \sigma_{meas}^{2}\left(k\right) & 0\\ \;0 & \sigma_{meas}^{2}\left(k\right) \end{array}\right]\\ \;K=P_{k+1|k}H^{T}{\left(HP_{k+1|k}H^{T}+R_{k}\right)}^{-1}\\ \;{\hat{x}}_{k+1|k+1}={\hat{x}}_{k+1|k}+K\left(z_{m}-\hat{z}\right)\\ \;P_{k+1|k+1}=\left(I-KH\right)P_{k+1|k}{\left(I-KH\right)}^{T}+KR_{k}K^{T}. \end{array}$$

Other factors such the number of tracked objects near the center of the fovea as well as the size of the objects could also affect the accuracy of the measurement, but are not considered in this paper.

## 6. Application Demonstration

**Figure 20:**
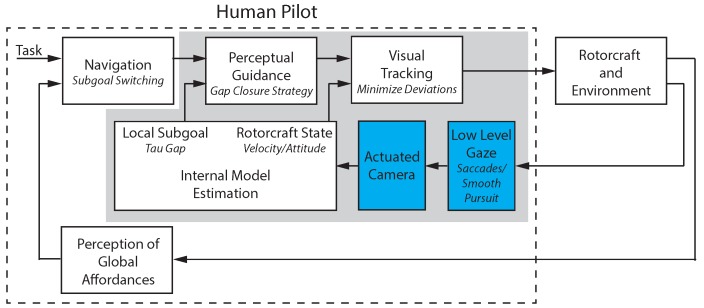
The components implemented for each example application are highlighted. The blue boxes are the components for the actuated camera and the blocks inside the gray boundary are implemented in the augmented control example.

A significant aspect of the human visual experience is due to head–eye coordination. At the same time, gaze control mechanisms are closely involved in the guidance and control of movement. The video display in current teleoperation setups does not account for the natural head–eye interactions and therefore can adversely impact performance. This section investigates automating remote camera positioning based on the operator gaze behavior. The camera is mounted on an actuated gimbal that uses real-time gaze measurements to mimic human head movement. A second application example implements control augmentations to demonstrate how the gaze can be used as part of the vehicle control architecture. Figure 20 shows the components relevant to each of the two application examples. Implementation details for the highlighted components are provided in the following sections.

### 6.1. Background and Overview

Teleoperation systems and the issues related to their successful implementation have been studied extensively. Autonomous or semi-autonomous operations have made important progress, but for the foreseeable future human teleoperation will remain a critical modality, in particular for interactive tasks such as surgery or vehicle operation in complex environments.

#### 6.1.1. Application Overview

The present work focuses on teleoperation systems for remote-control tasks. Experiments are conducted using a miniature rotorcraft as shown in Figure 3b. The teleoperation camera can be rotated to change the view of the environment or track the miniature helicopter during flight. The system uses a GoPro camera mounted on a tripod with a 3-axis motorized gimbal that provides controlled rotation of the camera to mimic head movements. A live video feed from the camera is shown on the teleoperation display. The pilot sits in front of the teleoperation display (facing away from the lab environment), and operates the rotorcraft using only the visual information from the display. The pilot’s gaze location on the display is captured using a gaze tracking device and is used in conjunction with models of the head–eye system to automate control of the camera orientation. The general approach could be used in other applications where visual guided motion is important, such as robotics, video games, or telesurgery.

#### 6.1.2. Related Work

Approaches for teleoperation have been proposed to help overcome problems resulting from a limited field of view (FOV). Voshell [86] developed a multi-camera system that provided the operator a wrap around effect in order to increase the FOV. In [87], FOV issues were investigated with the conclusion that performance could be optimized when the display and camera have the same FOV. Zhu [88] actuated a camera based on gaze using a simple “move to the center” method that actuates the camera to keep the gaze in the middle of the image. An approach using gaze as a control input overlaid boxes on the display that allowed the user to select actions by focusing the gaze on different areas [89]. Also relevant are applications of human head–eye models for control of a robotic head [90].

#### 6.1.3. Approach Overview

To date, limited research has been devoted to the investigation of the use of gaze as an integral part of the control strategy in teleoperation [7]. In [7], the coupling between gaze modes and human control actions was investigated. Experiments were conducted using a miniature helicopter while data about the vehicle state, control actions, and operator gaze were recorded. The relevant control information was extracted by decomposing the gaze into saccades and smooth pursuit and examining the gaze patterns. In the following, the understanding about the gaze interaction is used to optimize the teleoperation system. First, to optimize the live video display and second to optimize the control modality. The goal is to provide natural experience and exploit the head–eye control mechanisms. The effectiveness of the approach is evaluated comparing the operator workload for different teleoperation configurations.

### 6.2. Gaze-Mediated Camera Control

**Figure 21:**
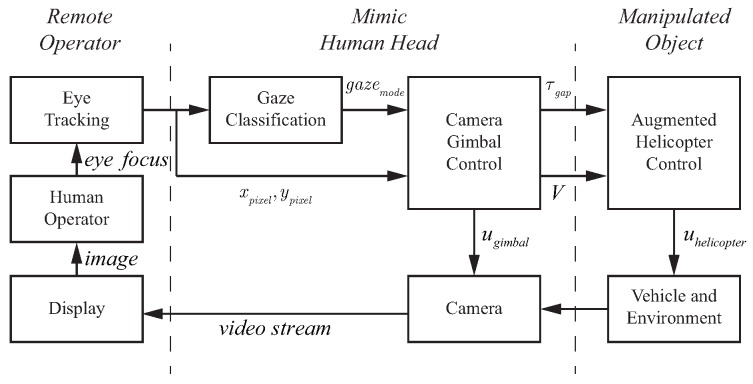
Components of the gimbal control architecture.

To mimic head movements, the approach shown in Figure 21 was utilized. As seen in the figure, the systems for gaze classification and gimbal control need to be defined. In addition, the gimbal control model needs to account for the different modes of gaze operation, i.e., saccades and smooth pursuit. This section describes experimental results and modeling of the components highlighted in blue in Figure 20.

#### 6.2.1. System Overview

The block diagram of the gimbal control architecture is shown in Figure 21. The key components are the eye tracking device, the gaze classification algorithm, and the gimbal control system. The eye tracking device extracts the location of the operator’s focus of attention on the teleoperation display with the gaze classification algorithm from Section 5.1.2, determining whether the gaze is currently in a fixation, saccade, or smooth pursuit mode. The gimbal control system takes the gaze location and mode to generate control signals that manipulate the camera orientation to conform to natural head movements. In addition, the gimbal control system also generates information for the control of the rotorcraft in the form of the rotorcraft velocity (*V*, the estimated value of the human operator efference copy of helicopter velocity) and the gap to close ($\tau_{gap}$) with respect to the current subgoal for the task.

#### 6.2.2. Gimbal Control

Gimbal control that mimics the human head–eye system requires different control approaches for the low-speed visual tracking of smooth pursuit and the high-speed gaze motion of a saccade. The appropriate gimbal control model is activated based on the current gaze mode (saccade or smooth pursuit). This section describes the gimbal control architectures implemented for generating saccades and performing smooth pursuit that emulates the human head.

##### Control for Saccades and Fixation

**Figure 22:**
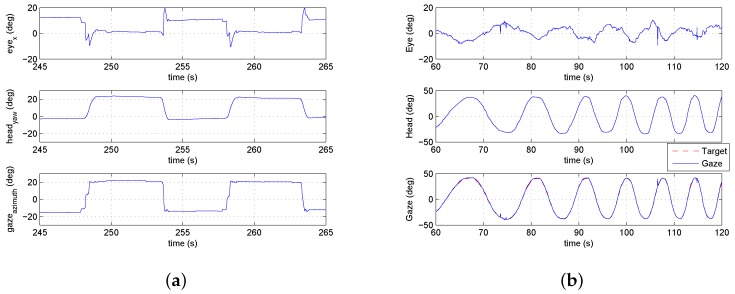
Experiments for saccades and smooth pursuit gaze tracking. (**a**) Head–eye-gaze time history for the saccade experiment using eye tracking glasses to follow a moving laser point (in the body frame). (**b**) Head–eye-gaze time history for the smooth pursuit experiment using eye tracking glasses to follow a moving laser point.

To model the human head–eye system response to gaze shifts of varying size, an experiment was conducted using a laser pointing system. The laser point on the ground was controlled to produce steps of varying size that the human subject was instructed to follow with their eyes. An example of the resulting eye, head, and gaze patterns is given in Figure 22a. As seen in the figure, the eye has an initial fast response to a gaze shift while the head responds slower. The combined head–eye motion generates a clean and repeatable gaze shift.

**Figure 23:**
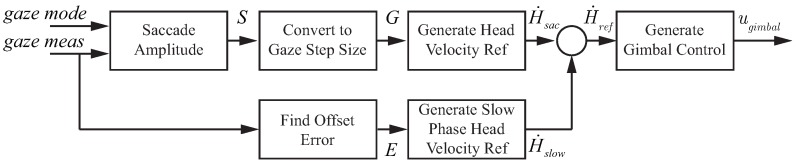
Block diagram showing the configuration of the saccade and fixation gimbal controller.

The model used to control the gimbal during gaze shifts is shown in Figure 23. The model is based on [64] and generates a fast head velocity that is determined by the size of the gaze shift. In addition, the controller captures the slow phase response necessary to correct the head position at the end of the gaze shift. The control of each dimension (azimuth and elevation) is treated independently with Figure 23 showing one dimension.

The size of the gaze shift (*S*) is identified from the amplitude of the initial eye saccade. In [64], it was shown that the amplitude of the eye saccade and the size of the gaze shift are linearly related. Based on experimental data, a linear model was identified to convert the eye amplitude to a gaze step size (*G*) as follows

(15) G=1.8S−1.17.

The linear fit had an $R^{2}$ value of 0.81 and is used to convert the eye amplitude to a gaze step size. To generate the head velocity reference, the gaze step size is multiplied by a constant ${\dot{H}}_{sac}=1.25G$. The slow phase head velocity reference uses a constant gain to convert the distance of the eye from the center of the display into a corrective head velocity. The slow-phase head velocity reference is attenuated for large eye offsets to disable the correction during large gaze shifts.

##### Control for Smooth Pursuit

Experimental evaluation of the head–eye system operation in the smooth pursuit mode was conducted using the laser pointing system. In the experiment, a chirp signal was applied to move the laser point at increasing velocity along a trajectory that caused yaw movement of the head. An example of the resulting eye, head, and gaze patterns is given in Figure 22b. The figure shows that the eye and head coordinate to track the moving point with the head providing the majority of the motion.

**Figure 24:**
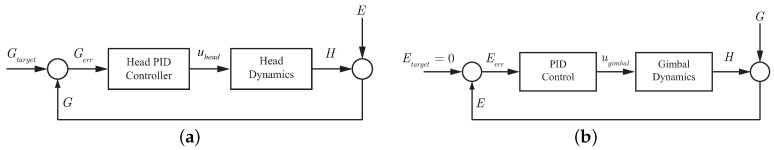
Block diagrams for smooth pursuit control. (**a**) Block diagram showing the structure of the head–eye control during smooth pursuit. (**b**) Block diagram showing the configuration of the smooth pursuit gimbal controller.

The smooth pursuit mode of head–eye coordination follows the model shown in [65]. The head control and dynamics for this type of system are shown in Figure 24a. In the system, the gaze error ($G_{err}$) is used in closed-loop control to drive the head along a trajectory that tracks the desired target. The combined head–eye motion provides the gaze.

From the experimental data collected during the experiment shown in Figure 22b, the frequency response was calculated for the open-loop from $G_{err}$ to *H* using the FRESPID tool in CIFER [78]. A transfer function was fit to the frequency response using the NAVFIT tool. The transfer function assumes a PID form ($K_{p}+K_{d}s+K_{i}s^{-1}$) for the control and second-order dynamics for the head. The objective for the gimbal control system is to match the dynamic response of the human head–eye system in order to provide a response that feels natural to the operator.

To implement gimbal control for the smooth pursuit mode, the block diagram from Figure 24a is modified to the form in Figure 24b. The assumptions used to make the changes are that the eye error ($E_{err}$) is equivalent to the gaze error ($G_{err}$), which holds true while the target is in the field of view and that the head control system functions to keep the eye centered on the display. A PID controller was implemented rather than a more advanced control strategy in order to match the existing model of human smooth pursuit found in [65]. Each dimension, azimuth and elevation, is treated as a separate loop.

#### 6.2.3. Experimental Results

This section presents the results from experiments conducted using the gaze-mediated teleoperation system.

##### Environment Sensing with Saccades

**Figure 25:**
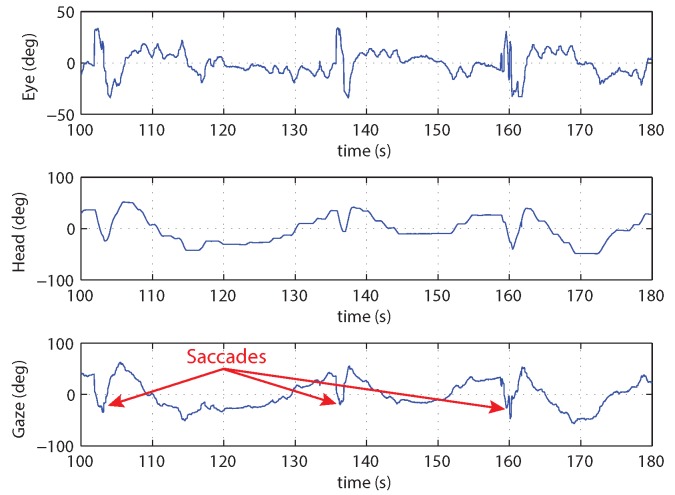
Saccades generated by the eye tracking/gimbal system.

When a saccade is detected, a fast gimbal motion is executed to reposition the camera to point at the area of interest. An experiment was conducted with a single starting location and multiple possible goal locations (for details see [7]). While hovering over the start position, the operator was instructed to fly to one of four possible goal locations. Due to the operator’s uncertainty about the goal locations, saccades were generated to quickly verify the positioning of the goal before beginning the maneuver. Figure 25 shows an example of the head–eye-gaze during the experiment. The figure shows three trials of the pilot flying the vehicle from a start location to one of the possible targets and then returning to the original position. At the start of each trial, a saccade is generated when the pilot is informed of the next target.

##### Performance Evaluation

This section compares the proposed teleoperation system with alternative configurations. The first configuration used a camera with a fixed field of view while the second option had an additional operator manually controlling the camera motion. The experimental task had the helicopter operator hover the vehicle over a location marked on the floor. Once a stable hover was achieved the operator maneuvered the vehicle to hover over a second marked location (see Figure 7). The task of moving between the two locations was conducted 20 times. The experiment was executed at slow, medium, and fast speeds for each of the teleoperation configurations.

**Figure 26:**
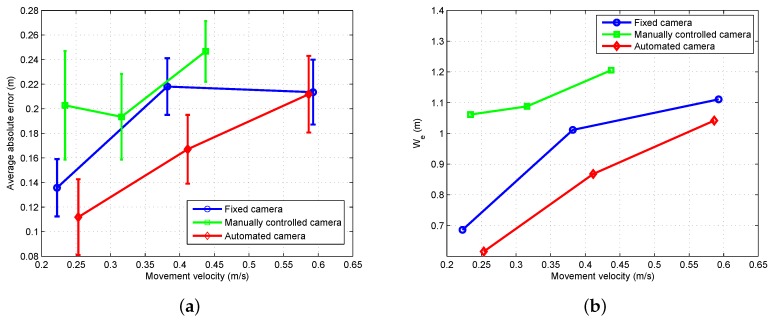
Comparison of task difficulty for different teleoperation configurations. (**a**) Error versus speed comparison. (**b**) Effective width versus speed comparison.

The accuracy metric defined in Section 3.3.3 was used to analyze performance. The experiment defined the movement velocity and the teleoperation configuration as the independent variables with $W_{e}$ as the dependent variable. The expectation was that plotting the movement velocity against $W_{e}$ for the different configurations would generate approximately linear results with the more challenging teleoperation configurations having higher $W_{e}$ values. Figure 26 shows the results for the three configurations and three movement speeds. Based on the analysis, the proposed approach was the easiest for the operator while the manually operated camera proved the most difficult. The hypothesis for the narrower range of speed for the manually controlled camera is that the pilot had more difficulty perceiving the helicopter motion than for the other two configurations. The pilot reduced the speed for the medium and fast cases to compensate for impaired perceptual abilities.

### 6.3. Augmented Flight Control

A second application example builds on the automated camera gimbal from the previous section. In addition to controlling the camera, processes are implemented to estimate the state of the vehicle and task elements using gaze, generate perceptual guidance trajectories, and perform visual tracking. Experiments using a step task demonstrate the system capabilities. Figure 20 shows the components implemented inside the gray box.

#### 6.3.1. Implementation of Augmented Control

The procedure for automating the camera gimbal was presented in Section 6.2. The implementation of the remaining components from Figure 20 follows directly from the results of Section 4 and Section 5. The internal model for estimation of the local subgoal and vehicle state was described in Section 5.5. The approach for generating a reference velocity based on the concept of a tau guide was defined in Section 4.2. Finally, the visual tracking component identified in Section 4.1 provided the feedback and feed-forward functions. The only change to the control models was a slight reduction in the feedback gains due to the 120 ms delay in the video feed of the teleoperation system. The individual elements were implemented as real-time processes that were integrated into the ROS software environment.

#### 6.3.2. Experimental Results

A set of flight experiments was conducted using the teleoperation system with augmented control. The pilots were asked to perform the step task from Section 3.2.2. At the beginning of the task, the pilot was instructed to achieve a stable hover over the initial location and then signal the start of the motion by toggling a switch on the joystick. The augmented control system would then generate a control signal to move the vehicle to the target location with the pilot having the capability to correct errors in the trajectory using the joystick lateral and longitudinal control inputs. In general, the majority of the motion was successfully handled by the control augmentation with the pilot only providing minimal corrective action around the target. It should be noted that the experimental results are meant to demonstrate the potential benefits of the model’s functional and structural characteristics. More experiments would be required to make precise claims about the performance characteristics.

**Figure 27:**
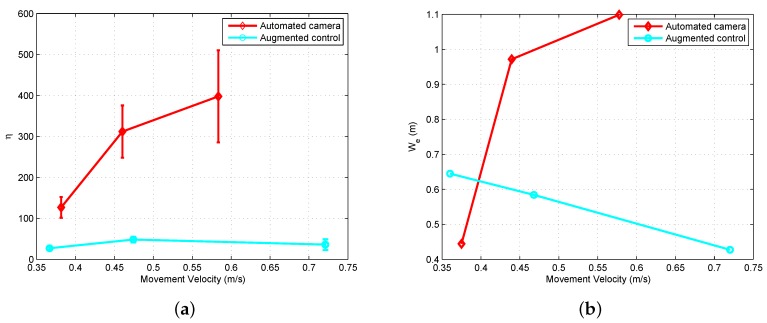
Comparison of the operator workload for different teleoperation configurations. (**a**) Workload versus speed comparison; (**b**) Effective width versus speed comparison.

Figure 27 shows a comparison between a system configuration that only automated the camera motion and a system with the full control augmentation. The workload metric was defined in Section 3.3.3 with the accuracy given by effective width $W_{e}$. As seen in Figure 27a, the augmented control configuration had a significantly lower workload than the automated camera configuration. This indicates that the control augmentation is able to successfully take over a large portion of the control action, thus reducing the workload on the pilot. Figure 27b demonstrates that the augmented control system also does well when comparing accuracy. The augmented control configuration has slightly worse accuracy at low speed when compared to the automated camera configuration, but as the speed increases the augmented control clearly performs better.

**Figure 28:**
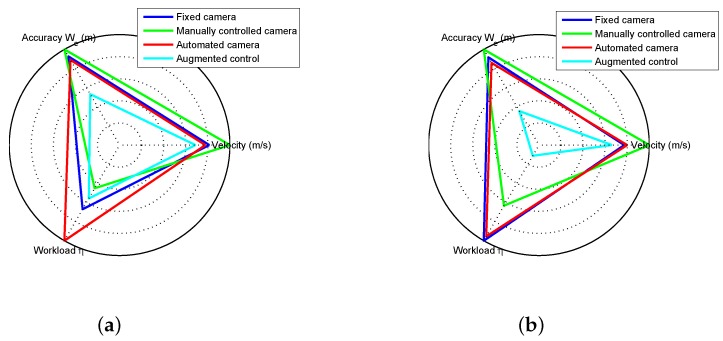
Comparison of speed, accuracy, and workload trade off for different teleoperation configurations. (**a**) Medium Speed; (**b**) Fast Speed.

In Section 3.3, a set of metrics for evaluating performance was defined based on “the big three” of speed, accuracy, and workload. Figure 28 shows the metrics on a single plot to make comparison between configurations easier. The configurations tested were a fixed camera, a manually controlled camera (by a second operator), the automated camera of Section 6.2, and the augmented control approach. Figure 28a,b show results for the step task when performed at medium and fast speeds. In the figures, the dimensions are scaled such that lower values (accuracy, velocity, and workload) are considered better performance. At medium speed, the augmented control configuration had the best accuracy and velocity and second best workload (behind the manually controlled camera). For fast speed, the augmented control provided the best performance for all three metrics.

## 7. Conclusions

In this paper, we presented a general approach to modeling a human operator’s control and guidance response in a task where the sensing and control is mediated by the human visuo-motor system. The proposed system model captures the human–vehicle–environment interactions focusing on the role of gaze dynamics. A multi-loop architecture organizes the control response into three levels. The lowest level acts as a simple control element to perform visual tracking. The next level, perceptual guidance, employs learned visuo-motor patterns to close gaps between the perceived state and the desired state. The final level considers the task and environment to determine the current subgoal. This paper characterized the first two levels of the multi-loop control architecture and identified specific models for the perceptual guidance and visual tracking components. With respect to perception, the visual information extracted by the human operator is registered in a body centric spherical reference frame that corresponds to human visual perception. Decomposition of gaze measurements into smooth pursuit and saccades provided the information necessary to estimate the state of the vehicle and task elements.

Models were identified for the perceptual and control components of the multi-loop architecture. The pilot’s control response was characterized by identifying models for the perceptual guidance and visual tracking blocks. Tau theory provided the basis for modeling perceptual guidance. The concept of a tau guide was utilized to generate reference trajectories by matching the tau guide-generated control with the experimental control response. At the visual tracking level, we identified models for feed-forward and feedback control elements. The feed-forward element was found by inverting a simplified version of the helicopter dynamics. System identification techniques fit the control parameters for the feedback component. Gaze was utilized as the primary sensory mechanism for measuring vehicle state and task elements. We demonstrated how the gaze patterns can be decomposed into smooth pursuit and saccades. These visual cues were analyzed to determine the primary visuo-motor control mechanisms in the multi-loop human control system. We showed that the smooth pursuit gaze behavior provides a measure of the rotorcraft state necessary for stabilization and regulation. The saccades, on the other hand, measure the gap to the goal location, which is consistent with tau theory. Using the information extracted from gaze, we designed an estimation model that tracked the vehicle state and task elements.

Finally, we applied the models for human perception and control to a real-world problem. The first example application utilized gaze to automate positioning control of a remote camera based on models of the human head–eye system. The architecture implements control of the remote camera that mimics human head movement and consequently is more natural for the operator. We evaluated the application using a version of Fitts’ Law that showed that the system exhibited improved performance in comparison to a fixed or manually operated camera. A second example application augmented the control system to aid the pilot while still allowing pilot input. The augmented control example demonstrated better performance for the accuracy, speed, and workload metrics when compared to the other teleoperation configurations (a fixed camera, a manually controlled camera, and a camera with automated motion).

## Figures and Tables

**Table 1:** Human-in-the-loop trade-offs [8].

Humans Are Better at	Machines Are Better at
Stability in control response	Fast control response
Adaptation to changing environments	Repetitive and precise tasks
Pattern recognition and processing large amounts of data	Processing information
Inductive reasoning	Deductive reasoning
Performing when overloaded	Multi-tasking
High-level goal selection and planning	
